# Software Framework for the Creation and Application of Personalized Bone and Plate Implant Geometrical Models

**DOI:** 10.1155/2018/6025935

**Published:** 2018-10-10

**Authors:** Nikola Vitković, Srđan Mladenović, Milan Trifunović, Milan Zdravković, Miodrag Manić, Miroslav Trajanović, Dragan Mišić, Jelena Mitić

**Affiliations:** Faculty of Mechanical Engineering, University of Niš, Niš, Serbia

## Abstract

Computer-Assisted Orthopaedic Surgery (CAOS) defines a set of techniques that use computers and other devices for planning, guiding, and performing surgical interventions. The important components of CAOS are accurate geometrical models of human bones and plate implants, which can be used in preoperational planning or for surgical guiding during an intervention. Software framework which is introduced in this study is based on the Model-View-Controller (MVC) architectural pattern, and it uses 3D models of bones and plate implants developed by the application of the Method of Anatomical Features (MAF). The presented framework may be used for preoperative planning processes and for the production of personalized plate implants. The main idea of the research was to develop a novel integrated software framework which will provide improved personalized healthcare to the patient, and at the same time, provide the surgeon with more control over the patient's treatment and recovery.

## 1. Introduction

Human skeletal system is often affected by some pathological processes, injuries, and fractures. In many cases, it is necessary to perform a surgical intervention, sometimes quite urgently. For the surgical intervention to be successful, it is particularly important to prepare a good preoperative plan and to ensure that adequate implants are provided. Computer-Assisted Orthopaedic Surgery (CAOS) [1] defines a set of techniques that use computers and other devices for planning, guiding, and performing surgical interventions, and it can be developed for various platforms [2]. The important components of CAOS are accurate geometrical models of the affected human bone and personalized 3D models of plate implants, which can be used in preoperational planning or for surgical guiding during the intervention [3]. A higher geometrical and anatomical accuracy of the applied geometrical models can improve pre-, intra- and postoperative procedures, and as a consequence, patient's recovery. In general, geometrical models of human bones can be created by the application of two different approaches [4–8]. The first approach implies the application of medical imaging technologies, like computed tomography (CT), in order to provide 3D geometrical models of human organs. Such models can be created in three general ways: by the application of specialized software, which is a part of a medical scanner (e.g., Vitrea); postprocessing of medical images in medical-oriented CAD programs (e.g., Materialise Mimics); postprocessing in one of the CAD software packages (e.g., CATIA). One of the main drawbacks of this approach is the inability to create a model of a whole bone. That is the case when the bone scan is incomplete due to illness (osteoporosis, arthritis, cancer, etc.) or trauma (multiple fractures, crushed bones, etc.) or when the quality of medical images is not good enough. The second approach for creation of 3D geometrical models of bones or bone segments is based on the predictive geometrical or statistical model of bones and data obtained from medical images [4,5,7–10]. For example, these models can be created by the application of statistical methods or FFD (free-form deformation) techniques. Geometric entities of predictive models are described by mathematical functions, whose arguments are morphometric parameters that can be read from medical images. By using this approach, it is possible to create a 3D geometrical model which corresponds to the patient's bone, i.e., personalized models. Possible disadvantages of this approach are the inability to read all parameters (for the same reasons as in the first approach), insufficient number of parameters involved in predictive functions, as well as inadequately defined parameters.

One of the possible applications of geometrical models of human bones is for the internal fixation. Internal fixation is a surgical technique in which implants (plates, rods, pins, etc.) are placed inside of the human body. For bone fixation and reduction, plate implants are widely used. There are several types of these implants, but most common are dynamic compression plates (DCP) and locking compression plates (LCP). DCP [11, 12] improved healing processes by eliminating the need for external immobilization and providing more stability to the fixation assembly. In order to fulfil their function, DCPs have to be mounted onto the periosteum (the tissue that surrounds bone surface) and should be pressed onto the bone to achieve stability [12–15]. As a consequence, blood supply to the bone can be interrupted, and cortical bone porosis can appear at the site of implant placement. A new plate design, the limited contact-dynamic compression plate (LC-DCP) [12], was introduced in order to reduce cortical necrosis. In today's practice, locking compression plates (LCP) are most common [14]. These plates provide locking (locking screws) and nonlocking function, which means that they can combine properties of both the mentioned plates (DCP and LC-DCP). The precontouring of plate shape, i.e., the personalization of plate shape is a common operation which can be done before or during the surgical intervention. The need for precontouring is generally determined by a type of plate and bone disease. For example, reconstructive plates [16] require precontouring in order to achieve mechanical stability, but for LCP with locking screws it is not required, because locking screws provide stability to the fixation system. Regardless of the precontouring requirement, the possibility to adjust a plate shape is a good software feature, because it gives the surgeon more flexibility in planning the orthopaedic intervention, and the research presented in this paper provides that functionality.

Software platforms used for preoperative planning and guiding in orthopaedics have been developed for many years, and there are many different solutions. There are commercial solutions like Materialise Mimics and OthoView (https://www.materialise.com/en/medical/), or Osirix (https://www.osirix-viewer.com/) [17] which uses medical images in appropriate format (DICOM) to plan a surgical intervention or to analyse the patient's health state. There is a possibility for surgeons to use software which is distributed with the scanning systems like Vitrea (http://www.vitalimages.com/vitrea-vision/vitrea-advanced-visualization/) or Vesalius3D (https://www.vesalius-3d.com/), which enable direct processing of the 2D scanned images. Open-source or free solutions are also used, like RadiAnt (https://www.radiantviewer.com/) or MicroDicom (http://www.microdicom.com/) which can be used for the reconstruction of DICOM images and creation of a 3D model of the scanned tissue. All of this software is based on the direct implementation of medical images obtained from medical scanning devices [18, 19]. In these cases, when scanned data of a human organ are incomplete, the proper reconstruction of their missing part or the creation of a personalized plate implant model is very complex, and therefore, the accuracy is questionable [4, 6, 20].

In order to improve pre-, intra-, and postoperative procedures, a new software framework based on Model-View-Controller (MVC) architectural pattern [21] was developed. The software framework is named Personalized Orthopaedic Model-View-Controller (POMVC) framework. The POMVC framework represents integrated software environment which uses and improves Method of Anatomical Features (MAF) [5, 7, 8] procedures and algorithms for the creation of personalized 3D models of human bones and plate implants. Integrated means that the framework includes individual software components used for the creation of 3D models and connects them in one functional platform. By the authors' knowledge, there is no similar kind of framework currently applied for the creation of bone and plate models or for preoperative planning. Possible benefits of POMVC framework application can be creation of complete bone models and/or plate implants even in the cases when the input bone data is incomplete; shorter time of surgical intervention due to better preoperative planning; manufacturing of personalized plate model by the application of additive manufacturing or high-speed machining; generation of bone and plate model databases, which can be used for further research and others.

The paper is structured as follows: the first section of the paper describes bone samples used for the research. In the next section of the paper, descriptions of the MAF and plate 3D models are presented. Thereafter, the POMVC framework and its components are described in detail. In the last section of the paper, the application of the POMVC framework for a real clinical case is presented.

## 2. Materials and Methods

In order to demonstrate the methods and the POMVC framework, human tibia and mandible samples were used. The samples were scanned by 64-slice CT (MSCT) (Aquillion 64, Toshiba, Japan), with a resolution of 512 × 512 px and a slice thickness of 0.5 mm. The same samples were used as the ones presented in [7, 8].

### 2.1. Research Concept

In this section of the paper, first a short introduction to the methods developed by the authors of the research, which represent the foundation for the research presented in this study, is provided. Next, a description of the software framework, its components and application in real medical case is shown.

#### 2.1.1. Method of Anatomical Features (MAF)

MAF is a method developed by the authors of this research, and it introduces a new approach for the creation of geometrical models of human bones. The main objective of MAF application is to provide complete 3D geometrical models of human bones and bone fragments, even in the cases where the input data of a patient's bone are not complete, due to the bone illness, fracture, or some other trauma. In general, two different types of output models can be created by MAF:

- 3D geometrical models: these models are standard polygonal, surface, and volume models which have been used in CAD for many years. They are created by the application of standard CAD technical features in CAD software packages, on the input models created by medical imaging methods. The procedure for creating such models consists of several processes: CT scanning of the patient; segmentation of medical data in medical software (e.g., Mimics); transformation of geometrical data in medical software to an appropriate format (e.g., STL) for CAD; importing 3D model into CAD software; model manipulation and transformation in CAD software; and creation of an adequate 3D model (polygonal, surface, solid, etc.). The procedure is described in more detail in [5, 7].
- Predictive (parametric) models of human bones: these models are statistical models formed over the input set of bone samples. They are defined as point cloud models, which represent an approximation of the human bone boundary surface. Each point coordinate (*X*, *Y*, and *Z*) in the point cloud is defined by an individual parametric function. Currently, multilinear regression is applied for the creation of parametric functions, and the used algorithm is presented in Equation (1) and defined in [24].

(1) $$\begin{array}{c} X=\left[\text{ones}\left(\text{size}\left(d_{1}\right)\right)d_{1}d_{2}d_{3}d_{4}\right],\\ A=X^{\prime }*X,\\ K=\mathrm{inv}\left(A\right),\\ B=\frac{A}{X^{\prime }*X_{\text{coord}}},\\ M=X*B, \end{array}$$

where *X*_coord_ is the vector of X coordinates defined for the input set cloud of point; *d*_*i*_ are the morphometric parameters (four); *B* is coefficient vector; and *M* is the vector of the calculated values (multilinear regression).

Parametric function is a function whose arguments are morphometric parameters. Morphometric parameters are dimensions which can be acquired (measured) from medical images by using adequate software, like Materialise Mimics, 3D doctor (http://www.ablesw.com/3d-doctor/), or GIMP (https://www.gimp.org/). By the application of individually defined values of morphometric parameters, these models can be customized to the geometry and morphology of a human bone of a specific patient. Currently, the process of acquiring parameter data is done externally by using medical or other image processing software, but the intention is to create in-house software solution which will be integrated into the POMVC. After the point cloud personalization, the model can be further processed in any CAD application, and other types of 3D geometrical models can be created [5, 7, 8].

It is important to note that parametric models are used in clinical cases when 3D data of human bone are missing (e.g., due to tumour, osteoporosis, and complex fractures), and so a complete 3D model cannot be created. By using medical software (e.g., Mimics and 3D Doctor) or X-ray images, a surgeon can acquire values of measurable morphometric parameters by using standard techniques [1–3] and apply them in parametric functions by using the POMVC framework. As the result of the process, a complete point cloud model of the bone is created, despite the lack of input data. Further description of the process for the creation and application of parametric models can be found in [5, 7, 8].

#### 2.1.2. Personalized Plate Implants

Personalized plates are based on three developed 3D models of the plates presented in [22, 23]. These models are as follows:

- Solid model of the personalized fixator. This model is a classical CAD model which can be used for the fixator manufacturing by the application of classical manufacturing or by the application of additive manufacturing processes.
- Optimal parametric model (OPM), which is based on the median bone geometry. This is a solid model whose dimensions can be changed according to the geometry of a specific bone. The median bone geometry is defined for the input set of bone samples, which defines a group of people for whom the fixator is applicable. This means that the fixator can be used for the patient whose bone geometric characteristics fall within the group.
- Parametric points model (PPM), which is based on the parametric surface model of the human bone developed by the application of MAF. The contact surface between the fixator and the bone is defined by the points whose coordinates' values are defined as parametric functions [5, 7, 8].

It is important to note that OPM and PPM models are developed by the authors of this research and described in [22]. The geometry of this fixator models can be adjusted in such a way that they can be used as DCP or LCP fixators. The shape is already precontoured, and the holes for screws can be adjusted to achieve screw locking.

#### 2.1.3. Description of the Software Framework

The developed software framework is based on MVC architectural pattern, and it can be applied for the creation of personalized 3D bone and plate models and for preoperative planning in surgery. The POMVC framework is composed of three main components: Model, View, and Controller, which are standard entities of any MVC software architecture [21]. The role separation is not strict, yet, each component can have multiple roles, which is influenced by the requested outcomes from the software and direct implementation. The POMVC framework represents integrated software environment which uses and improves Method of Anatomical Features (MAF) [5, 7, 8]. The integrated framework consists of several software components which are connected into one functional platform. In the current version of the POMVC framework, included software components are MS Excel, MATLAB, and CAD software (CATIA and/or Solid Works). All components are included as commercial or trial versions with adequate licenses. There are several reasons why this framework was developed in this way, and the most important reasons are as follows:

- The presented MVC architecture is very scalable, easily maintainable, and upgradable [21]. The system's components can be replaced by using software which is appropriate for the selected institution. The possibility to replace one of the components with some other open-source or in-house-developed solution adds great opportunity for further research and customization.
- The system's open architecture enables application of different algorithms for 3D model creation. This means that meshing of personalized cloud point models can be done by using a selected algorithm, like Delaunay triangulations, in custom-made applications, or by using specialized CAD software. Currently, meshing is done in commercial CAD component of the system (CATIA), but in the next versions of the POMVC framework, open-source solutions with enabled scripting will be used, like Blender (https://www.blender.org/) or FreeCAD (https://www.freecadweb.org/).
- Algorithms for the creation of parametric 3D bone models, originally created in MAF, are improved. Until the development of the POMVC framework, personalized bone 3D models were created by the application of parametric functions with strictly defined number of morphometric parameters [5, 7, 8]. POMVC enables application of parametric functions with variable number of morphometric parameters. This means that if a surgeon cannot acquire values of all defined morphometric parameters, which is the case with the missing bone data, then he can provide only morphometric values which can be acquired from medical images, or he can provide a minimal number of parameters which are adequate for the specific case. Based on the input data, POMVC will apply an adequate algorithm for the creation of personalized point cloud. The result may be a less accurate 3D model, yet, it will be a complete 3D personalized model of human bone.
- Until the development of POMVC, plate and bone parametric models were created and used directly in CAD software, with strictly defined procedures [5, 7, 8]. This means that a surgeon or a diagnostician needs to know specialized software features in order to create or manipulate 3D models or to engage a designer who will provide support. By introducing POMVC, application of parametric models is much simpler, because interface for entering morphometric data and parametric model manipulation can be adjusted to the surgeon needs. Of course, the presence of a designer is still recommended, but not mandatory. It must be noted that the POMVC framework still enables the application of individual components for specific cases, as it is in the clinical case that will be presented in the later section of the paper.
- Bidirectional connection between components included in the POMVC framework is established. This means that bone and plate models created in the framework can be used for improving parametric models by enlarging the input set (CAD to Excel), forming the database of bone and plate models (CAD to database) which can be used for further statistical or other AI processing, etc.

It is important to define how MAF is integrated with the POMVC framework. MAF is a method with many developed procedures for the creation of personalized 3D models of human bones and plate implants. It contains algorithms (1), procedures, rules, and other elements which must be implemented in order to fulfil specific goals. The main question is how these elements are integrated or represented in the POMVC framework. The construction of personalized 3D geometrical models based on complete bone data requires application of just one component of the framework and that is CAD software. The input for this procedure is a 3D model created in medical imaging software, e.g., Mimics. Application of parametric bone model requires a different approach. First, the algorithm for the creation of a personalized point cloud model of a specific bone or bone part must be stored in some kind of a database system. The POMVC uses textual files and relational database. Each algorithm is defined by Model (e.g., Module in VBA or m file in MATLAB), and based on the user input, the controller component decides what model will call, i.e., perform business logic. As already stated, algorithms cannot work without data (point data, morphometric parameters, and plate model parameters) so it is important to enable input of data which will be forwarded to Model component. This input is done through view Component and its UI (e.g., Excel cells or text fields, CAD UDF (user-defined feature) or plain parameters). Based on the requirements and input data, the framework creates adequate bone and plate models or enables the surgeon and/or designers to do additional modelling processes.

**Figure 1:**
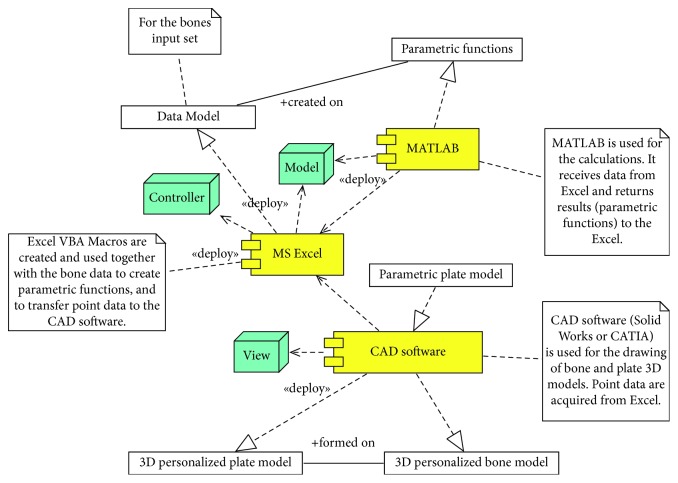
UML component diagram of the POMVC architecture.

In order to provide visual representation of the software components and their mutual influence, UML component diagram is created and presented in Figure 1. The description of individual components included in the current version of the POMVC framework is as follows.

*(1) MS Excel (Controller and Model)*. This component is an entry point for the POMVC. This component contains input fields for entering point data, values of morphometric parameters acquired from medical images, and for inserting plate parameter values for the plate personalization. It contains VBA macros which do calculations, call other components, and forward required data to them. These macros are as follows:

- Macro which contains the algorithm for the creation of parametric model of a specific bone based on the input bone samples and measured point data. As it has already been stated, a parametric bone model is created based on the input bone samples and the applied multilinear regression. The multilinear regression algorithm (1) is stored in Excel Module and in external “m” textual file. This macro can create parametric functions based on a specific number of input morphometric parameters, i.e., it can create “variable parametric models.” In order to create a specific parametric model, the textual file is transferred to MATLAB by using VBA subroutines and OLE (Object Linking and Embedding) technology, and calculations are performed externally. After the calculations, new point data (*X*, *Y*, and *Z*) are returned to Excel and placed in an adequate table cell. Created parametric functions are stored in external textual files for possible later applications, like further research or statistical processing. After the completion of macro, the user has an insight into each point coordinates for every point in a personalized point cloud, because they are presented in tabular format, in Excel.
- The second macro is used for transferring point data to CAD software. This is done by calling CAD software through OLE technology and applying adequate functions. Transferred points are used as the basis for the calculations of interpolated spline curves in CAD Software and for the creation of personalized bone surface and solid models. If there is a requirement, further model processing can be done directly in CAD software.

There is another specific function which Excel provides, and that is the ability to store parameters' values defined for CAD parametric plate model, i.e., it represents design table for the specific plate model. This is an excellent function which can be used for a quick creation of personalized plate model for the specific patient, when a 3D model of the bone is available.

*(2) MatLab (Model)*. This component is used for all calculations. In general, it can be considered as a Model and/or helper component. As already stated, multiple linear regression is currently used as a statistical method for tibia and mandible, but, other statistical and AI methods can also be applied. This component uses point and morphometric parameter data supplied by Excel to perform calculations on the basis of a defined algorithm (1). After the calculations, all results are returned to Excel.

Currently, for the mandible bone, neural network is also applied for the creation of a predictive model. An algorithm that covers this AI method will be included in the next release of the framework.

*(3) CAD Software (Model and View)*. CAD software is mostly used as View component of the POMVC. Point data for a specific bone are forwarded from MS Excel to CAD software and a geometrical model is formed by the use of specific technical features (e.g., Loft surface technical feature of Solid Works or multisection surface of CATIA). There is one additional function of this component which is important. This function enables the creation of a personalized plate geometrical model, based on the previously defined parametric solid model for a specific plate. In this stage of the POMVC, the creation of plate models is performed in CAD software, based on the values which are manually acquired from the created bone model [22]. These values are implemented as parameter values in the UDF (user-defined feature) manually, or by using a design table from Excel. If both the plate and bone models are created, then the CAD model can be used to form an assembly and to define possible manufacturing procedures through conventional manufacturing or by using additive technologies (3D printing).

#### 2.1.4. Process Description

**Figure 2:**
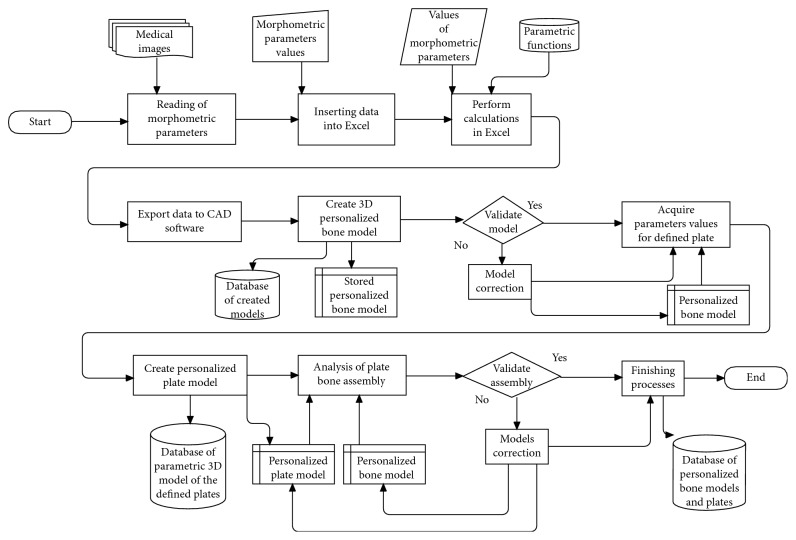
Flowchart diagram of the process for the creation of the personalized bone and plate 3D models.

**Figure 3:**
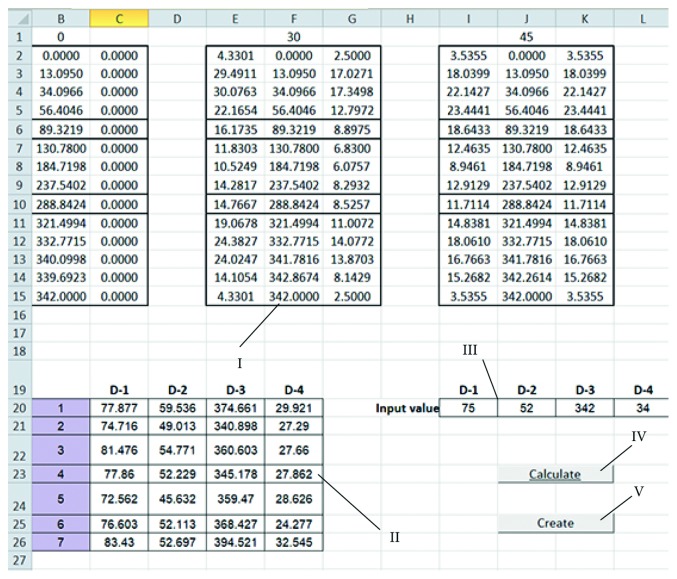
Point and morphometric parameter data presented in MS Excel. I : calculated point data for the specific bone; II : parameters defined for the input set; III : parameters for the specific bone; IV : first macro; and V : second macro.

A surgeon is the main user of the system. His role is to make important decisions and to perform surgical planning. The designer is a supporting user of the system. His role is to provide technical support for the surgeon and to improve and correct software system, according to the surgeon's recommendations. In order to better describe main processes of the proposed software framework, a flowchart diagram of the complete process for the creation of personalized plate for the specific bone and fracture is presented in Figure 2. The initial processes which are not included in the diagram are so-called preplanning processes, and they include patient admittance to the hospital and diagnostics of bone disease. Medical imaging is an important diagnostic process, and its output is the input for the first process in the system, which is “reading of morphometric parameters.” In that process, the surgeon acquires values of morphometric parameters from medical images; such are X-rays, or CT, in adequate software, or with other tools (etalons, measuring equipment, etc.). The acquired data are inserted in Excel tables, as already described, and calculations are performed. The surgeon only needs to enter the data, and all other tasks are done automatically. Calculated values are presented to the surgeon (Figure 3), and he only needs to press another button and start the second macro, which will trigger “Exporting data to CAD software” and “Creation of personalized Bone 3D Model” processes.

**Figure 4:**
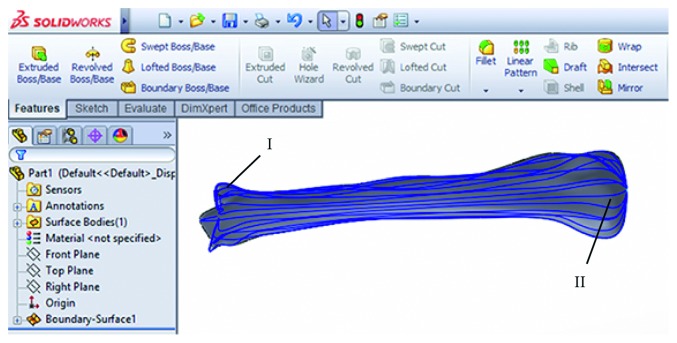
Tibia bone surface model created in CAD software component (Solid Works). I : interpolated spline curves; II : human tibia surface model created on the basis of interpolated spline curves.

**Figure 5:**
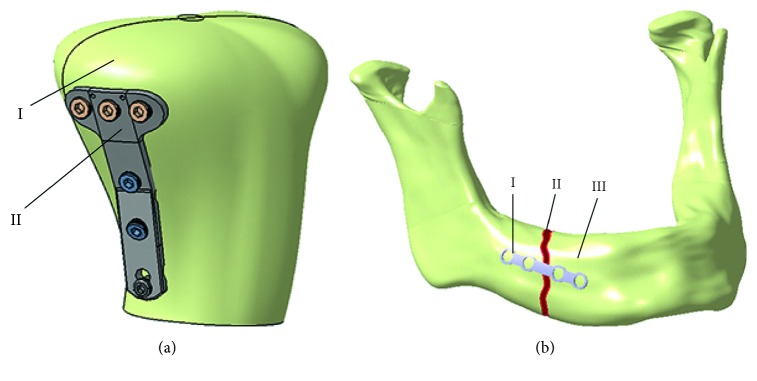
Assembly of personalized plate implant and bone 3D models: (a) proximal tibia bone (I) and modified cloverleaf plate (II); (b) customized reconstructive plate (I), mandible body fracture (II), and mandible bone (III).

The surgeon and the designer can analyse the created model, presented in Figure 4, and if there is a requirement for the bone model improvement, it can be done in CAD software. When the surgeon is satisfied with the created personalized 3D model of a specific bone, the next step is plate model selection. The surgeon can choose a plate model from the database of created plate parametric 3D models, or it can create a customized plate model based on the boundary surface of the bone [22]. In the current stage of the research, four parametric models of standard plates are created, three for long bones and one reconstructive plate for the mandible bone, but more will be developed. After the selection of a plate, parametric values are acquired from the bone model by the use of CAD technical features, and the plate model is modified accordingly. Parameters are defined differently for specific plates, and for the tibia bone and plate implant developed by Mitkovic, process of parameter acquisition is described in [22]. If there is a requirement for the model correction (e.g., parameter values are not measured right, the surgeon needs additional bending of the plate model, etc.), it is done in CAD software by the designer. When both the designer and the surgeon are satisfied with the 3D bone and plate models, final assembly is created, as presented in Figures 5(a) and 5(b). In the last step of the overall process (Finishing processes), 3D models are stored in a database for further research and application.

Considering the time required for the creation of 3D models, we can state that they can be created in a relatively short time, considering the described processes. When there are unacceptable deviations in models or the surgeon needs additional modifications, it can take a longer period of time to create the models, depending on the complexity of the problem. Also, if there is a requirement for model manufacturing, then the time for production depends on models' characteristics (geometry, shape, material, etc.) and on the applied manufacturing technology.

#### 2.1.5. POMVC Application in Real Surgical Case

*(1) Clinical Case Description*. The patient was a young (18 years) male with progenia. The main surgeon's requirement was to create personalized reconstructive plates which will provide fixation of mandible parts after surgery. The second important requirement was to avoid CT scanning for acquiring geometrical and morphometric data. For the purpose of fulfilling these requirements, the POMVC was applied. As already stated, the POMVC uses or creates geometric models of human bones and plates, and the only condition is to provide adequate input data (morphometric parameters, bone models, etc.) to the framework.

It is important to mention that the POMVC was used for a 3D mandible model creation in individual CAD component of the framework and for the personalized plate 3D model creation (Excel and CAD components were used). A short description of the mandible and a personalized plate 3D model creation process is presented in the next section of the paper.

**Figure 6:**
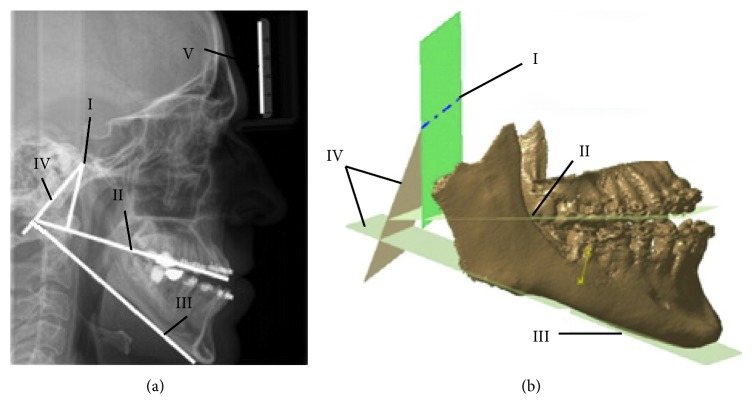
Assembly of personalized plate implant and bone 3D models: (a) X-ray image in LM plane—point of rotational axis (I), occlusion line (II), mandibular line (III), perpendicular lines (IV), etalon (V); (b) rotational axis (I), occlusion plane (II), mandibular plane (III), and perpendicular planes (IV).

*(2) Acquiring Input Data*. The patient was scanned by using an X-ray scanner (Figure 6(a)) and with Sirona SL Orthophos 3D device (Figure 6(b)). The X-ray scanning was performed with etalon included in order to properly scale measured values. X-ray and Sirona SL scanners were used together because Sirona SL field of view (FOV) size is less than required for the creation of a complete 3D model of mandible bone. The scanning FOV is 11 × 10 cm, and because of that, in the 3D medical image of the patient, both condylar processes were missing, as is presented in Figure 6(b). The lack of condylar process made it difficult to determine the position of the rotation axis of the temporomandibular joint on the 3D model. The rotation axis of the temporomandibular joint is significant because the mandible moves around it and rotates until the good occlusion is achieved. This is important because a proper plate shape can be created only when the mandible is properly positioned. Only then, a designer and a surgeon can choose valid points on the surface of the mandible model. To enable proper geometrical definition of the rotation axis, an X-ray image was used to acquire the missing geometrical and morphometric data.

In accordance with the anatomical and morphological characteristics of the mandible, two anatomical reference points are defined on the X-ray image in GIMP software: menton (ME) and gonion (GO) [8]. Menton is the lowest point on the mandibular symphysis, and gonion is the most inferior point of the mandibular angle. The horizontal (mandibular) line was obtained by connecting two anatomical points. The next step was to determine the position of the occlusion line. The occlusion line is an imaginary line that theoretically touches the incisal edges of the incisors and the tips of the occluding. Surgeons determined the position of the required points and elements on the 2D X-ray image: the point of rotational axis, ME, GO, incisal edges, and occluding tips. Next, lines which are perpendicular to the occlusion and horizontal line were created. These perpendicular lines are going through the point of rotational axis. The distances between the cross section of perpendicular lines with occlusion and horizontal line and selected points on the mandible were measured and scaled according to the etalon size. These distances were used to determine the position of the rotational axis in 3D. Thereafter, the position of the rotation axis of the temporomandibular joint (point) on the 2D medical image is determined, as presented in Figure 6(a). The same procedure was performed in 3D; only instead of lines, occlusion and horizontal planes were created. To determine the proper position of the rotational axis, distances which were measured in the 2D image were transferred to 3D. These distances provided positions of perpendicular planes on both the horizontal and occlusion plane (same as for lines in 2D). By using the intersection of planes perpendicular to the occlusion and mandibular plane, the rotational axis was determined.

*(3) The Creation of Plate Models*. In this specific case, good occlusion could be achieved by performing a cut on the mandible bone and by repositioning the parts. In cooperation with maxillofacial surgeons, the line of the cut is determined on the surface of the mandible polygonal model. The cut line is positioned in front of the seventh tooth on both the left and the right side. After cutting the polygonal model along the cutting line on the left and the right side, rotating and moving of the mandible is done in relation to the rotation axis of the joint. Having been rotated and moved, the mandible is brought into the appropriate position, in which the aesthetically pleasing appearance of the face is achieved. The most important goal was achieved, resulting in good occlusion.

Next, the mandible polygonal model was processed, and all anatomical points were transferred to Excel by using script in CATIA developed by the authors. By using this approach, a bidirectional connection was established between two components of the POMVC. Transferred anatomical points were used for enlarging the bone input set for the parametric model creation and for the following creation of the personalized plate models.

**Figure 7:**
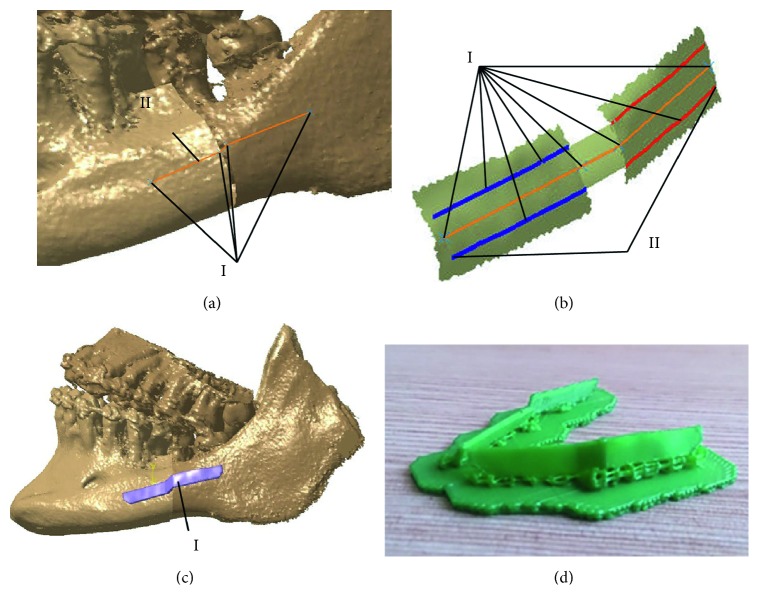
Creation of the personalized plates: (a) construction of reference line—anatomical points (I) and reference line (II); (b) surface extraction—anatomical points (I) and contour lines (II); (c) solid model of the plate (I); (d) printed models of the plate implants.

Following the maxillofacial surgeon's proposition, specific anatomical points from the set of defined points were selected on the mandible parts (Figure 7(a)). The line which connects these points determined the position of the plate (Figure 7(a)). The only thing left is to determine the shape of the plate which conforms to the boundary surface of the mandible. This was done by creating a surface model of the plate contact surface, by using anatomical points around the defined line, as presented in Figure 7(b). The plate model on the other side of the mandible was created in the same way. After the surgeons approved the surface model of the plate, the model was transformed to a solid model in order to prepare it for 3D printing (Figure 7(c)), by adding thickness of 3 mm. Physical models of both plates were printed on the CreatBot 3D printer and presented in Figure 7(d). Surgeons used these models for precontouring of reconstructive plates before surgery. Intervention was performed, and surgeons were very satisfied with the results, i.e., plates were precontoured perfectly. More information about the patient's recovery will be available in the following period.

## 3. Conclusion

Software framework presented in this research enables creation of personalized models of bone and plate implants customized to the geometry, morphology, and anatomy of the specific patient. Its scalable architecture and independent components provide a lot of possibilities for further improvement and adjustments. The main intention of the author of this research was to create a fully adaptable software framework which can be used independently in various institutions, for solving various problems in orthopaedics, and possibly other branches of surgery. Personalized medicine and various software solutions that support it are currently strongly applied in medical practice, and the presented POMVC brings additional possibilities to the field. The system can be used in medical education, clinical practice, and in all other fields where there is a requirement to provide an integrated system for the simulation and preoperative planning of surgical interventions in orthopaedics. Also, manufacturing companies can use the POMVC to create models of bones and plates by using conventional or additive manufacturing.

## Data Availability

The data used to support the findings of this study are available from the corresponding author upon request.
